# A study of Tuna (Katsuwonus pelamis) Nugget Effects on Cardiometabolic Risk Factors in Ischemic Cardiomyopathy at a Public Hospital in Lahore

**DOI:** 10.12669/pjms.42.6.16197

**Published:** 2026-06

**Authors:** Wajeeha Bashir Baig, Usman Naeem, Sajjad Ahmed, Ramesh Kumar

**Affiliations:** 1 Wajeeha Bashir Baig, Faculty of Allied Health Sciences, The University of Lahore, Lahore, Pakistan; 2 Usman Naeem, Department of diet and Nutritional Sciences, Faculty of Allied Health Sciences, Lahore, Pakistan; 3 Sajjad Ahmed, Department of Cardiology, Punjab Institute of Cardiology, Lahore, Pakistan; 4 Ramesh Kumar, Health Services Academy, Islamabad, Pakistan

**Keywords:** Cardiometabolic risk, Functional foods, Ischemic cardiomyopathy, Nuggets, Risk factor, Selenium, Tuna

## Abstract

**Objectives:**

To evaluate the effects of selenium-enriched tuna (Katsuwonus pelamis) nuggets on anthropometric, metabolic, and inflammatory risk factors in ICM patients in Pakistan.

**Methodology:**

A 12 weeks randomized controlled intervention study was conducted in Lahore, Pakistan from May 2025 - July 2025, 120 ICM patients were assigned to either a tuna nugget intervention group (n = 60) or a control group receiving standard dietary care (n = 60). The developed tuna nuggets were high in protein (31.93%), low in fat (1.75%), and enriched with selenium and antioxidants. Anthropometric measures, fasting glucose, lipid profile, and inflammatory markers (hs-CRP) were assessed at baseline and post-intervention. Mediation analyses evaluated the role of nutrient intake on cardiometabolic outcomes.

**Results:**

The intervention group showed significant im-provements compared to controls: weight (-1.8 kg, P = 0.034), BMI (-0.7 kg/m^2^, P = 0.028), fasting glucose (-6.7 mg/dL, P = 0.012), total cholesterol (-14.1 mg/dL, P = 0.021), LDL-C (-12.9 mg/dL, P = 0.018), HDL-C (+4.6 mg/dL, P = 0.009), triglycerides (-14.1 mg/dL, P = 0.031), and hs-CRP (-0.9 mg/L, P = 0.004). Mediation analyses indicated that protein, selenium, omega-3 fatty acids, fiber, and total antioxidant capacity significantly contributed to these effects, mediating 25–56% of the intervention’s impact. Sensory evaluation confirmed high acceptability of the tuna nuggets.

**Conclusions:**

Selenium-enriched tuna nuggets are a safe, palatable, and nutritionally effective dietary strategy to improve anthropometric, metabolic, and inflammatory risk factors in ICM patients. Functional food interventions like this offer a practical adjunct to conventional therapy, addressing cardiometabolic risk through targeted nutrient enhancement. Future long-term trials are warranted to evaluate sustained clinical benefits.

## INTRODUCTION

Ischemic heart disease (IHD) is now one of the leading causes of morbidity, mortality, and premature death worldwide, frequently progressing to ischemic cardiomyopathy (ICM) and heart failure.^1^ In low- and middle-income countries, including Pakistan, a “triple burden” of disease exists. Among these, NCDs, driven largely by modifiable lifestyle factors such as poor diet, physical inactivity, and obesity, have surpassed communicable diseases in prevalence.^2^ The recurrent hospitalizations and long-term management of ICM not only burden patients and families but also impose significant economic stress on healthcare systems.^3^ Metabolic disorders including Type-2 diabetes mellitus, insulin resistance, obesity, and dyslipidemia are well-established risk factors for cardiomyopathy and are collectively described as metabolic-associated cardiomyopathy.^4^

A deficiency of selenium, an essential micronutrient with anti-inflammatory and antioxidant properties, has been linked to worse cardi-ometabolic outcomes in patients with ICM.^5^ Despite this evidence, there is a scarcity of dietary intervention studies investigating selenium-rich functional foods, especially fish-based products, for improving CRFs and clinical outcomes in ICM patients.

Tuna (Katsuwonus pelamis) is a globally valued food source, renowned for its high-quality protein, omega-3 polyunsaturated fatty acids, and essential minerals most notably selenium in the forms of selenomethionine and selenocysteine.^6^,^7^ Lever-aging these unique nutritional properties, this study introduces a novel dietary intervention: selenium-enriched tuna nuggets specifically designed for patients with ischemic cardiomyopathy. By evaluating their impact on inflammatory biomarkers, lipid metabolism, and broader cardiometabolic risk factors, this research not only explores a promising functional food strategy but also addresses a critical gap in nutritional cardi-ology. The findings have the potential to transform adjunctive dietary therapy in ICM, offering an innovative, evidence-based approach to improve metabolic health and clinical outcomes in a high-risk population. By leveraging the nutritional benefits of tuna in a convenient, patient-friendly format, the study has the potential to inform public health strategies, clinical guidelines, and future research aimed at reducing the global burden of ischemic cardiomyopathy and associated metabolic disorders.

## METHODOLOGY

A 12 weeks randomized controlled intervention study was conducted in Lahore, Pakistan from May-July 2025 by recruiting 120 patients diagnosed with ischemic cardiomyopathy who were recruited from Punjab Institute of Cardiology, Lahore, Pakistan. Eligible participants were adults diagnosed with ischemic cardiomyopathy recruited from a tertiary care hospital, while individuals with severe comorbid conditions, dietary restrictions to fish products, or inability to comply with the study protocol were excluded. Participants (n=120) were randomly assigned in a 1:1 ratio to either the intervention group (tuna nugget supplementation, n=60) or control group (standard dietary advice, n=60). Randomization was performed using a computer-generated block randomization list with blocks of 10, stratified by sex and age. Allocation concealment was maintained using sealed opaque envelopes. Due to the nature of the dietary intervention, participants were not blinded, but outcome assessors and laboratory technicians remained blinded to group allocation. All laboratory analyses and clinical procedures were performed in accredited facilities under controlled conditions. Written informed consent was obtained from all participants before enrollment. The primary outcomes were changes in BMI, fasting glucose, LDL-C, and hs-CRP. Secondary outcomes included changes in total cholesterol, HDL-C, triglycerides, and dietary nutrient intake.

### Ethical Approval:

The study protocol was approved by the Institutional Ethics Committee of the University of Lahore (IRB: UOL/IREB/25/08/0005, Dated May 15, 2025).

### Tuna nugget preparation:

Tuna nuggets were prepared using canned skipjack tuna (Katsuwonus pelamis) and standardized food-grade ingredients, with an optimized formulation targeting high protein and low fat content, and processed under controlled hygienic conditions following a standardized protocol including rinsing and surface decontamination prior to further preparation.

### Ingredients and formulation:

According to Table-II The developed tuna nuggets demonstrate a higher protein-to-fat ratio compared to standard nuggets, making them suitable for patients with ischemic cardiomyopathy.

**Table-I:** Ingredient composition and proportions used in the formulation of tuna nuggets.

Ingredients	Quantity / Proportion
Tuna fish	90 g
Eggs	2
Corn starch	2 tablespoons
Garlic powder	0.5 g
Coriander powder	1 g
Curry powder	0.5 g
Cumin powder	1 g
Chili flakes & bread crumbs	As required

**Table-II:** Nutritional composition of tuna-based nuggets per 100 g.

Sample	Moisture (%)	Dry Matter (%)	Protein (%)	Fat (%)	Carbohydrates (%)	Total Ash (%)	Energy (kcal/100 g)
Tuna Nuggets (Developed)	34.61	65.39	31.93	1.75	30.75	0.93	266.59
Tuna Nuggets (Standard)	58.00	41.85	21.00	24.00	14.23	6.80	–
Tuna (Raw)	60.85	39.15	61.14	24.50	3.70	1.30	

Confirming homogeneity at baseline (all P > 0.05).

### Statistical analysis:

The distribution of continuous variables was evaluated using the Shapiro–Wilk normality test. For variables following a normal distribution, comparisons between the intervention and control groups were conducted using the independent samples t-test, whereas pre- and post-intervention comparisons within the same group were analyzed using the paired samples t-test. Variables that were not normally distributed were analyzed using the Mann–Whitney U test for between-group comparisons and the Wilcoxon signed-rank test for within-group analysis. Categorical data were assessed using either the Chi-square test or Fisher’s exact test, depending on the expected cell counts. Statistical significance was determined at a p-value less than 0.05. A pilot-tested, adapted standardized WHO global individual food consumption tool were used to ensure reliable anthropometric and lifestyle data collection.^8^-^10^

## RESULTS

Baseline characteristics of participants are shown in Table-III. No significant differences were observed between groups in BMI, blood pressure, lipid profile, fasting glucose, or lifestyle factors.

**Table-III:** Baseline characteristics of participants (n=120)

Variable	Intervention group (n=60)	Control group (n=60)	t / χ^2^	P value
Age (years, mean ± SD)	58.4 ± 6.7	57.9 ± 7.1	0.45	0.65
Gender, n (%)			0.20	0.65
Male	38 (63.3%)	36 (60.0%)		
Female	22 (36.7%)	24 (40.0%)		
BMI (kg/m^2^, mean ± SD)	28.3 ± 3.2	28.0 ± 3.5	0.46	0.65
Hypertension, n (%)	34 (56.7%)	32 (53.3%)	0.11	0.74
Diabetes mellitus, n (%)	21 (35.0%)	20 (33.3%)	0.03	0.87
Smoking, n (%)	18 (30.0%)	16 (26.7%)	0.11	0.74

The sensory evaluation of the tuna nugget intervention showed high acceptability among participants. Tale-IV. Mean scores (± SD) were 8.2 ± 0.5 for taste, 7.8 ± 0.6 for aroma, 8.0 ± 0.4 for texture, 8.1 ± 0.5 for appearance, and 8.2 ± 0.5 for overall acceptability, indicating that the product was well-received across all evaluated attributes.

After 12 weeks, the intervention group showed significant improvements in anthropometric and cardiometabolic outcomes compared to the control group. Table-V. Weight decreased from 78.3 ± 10.5 kg to 76.5 ± 10.1 kg (P = 0.034), and BMI reduced from 28.1 ± 3.5 kg/m^2^ to 27.4 ± 3.3 kg/m^2^ (P = 0.028). Fasting glucose declined from 112.4 ± 14.2 mg/dL to 105.7 ± 12.5 mg/dL (P = 0.012). Lipid profile improved, with total cholesterol decreasing from 198.2 ± 32.5 mg/dL to 184.1 ± 28.3 mg/dL (P = 0.021), LDL-C from 125.6 ± 28.1 mg/dL to 112.7 ± 25.4 mg/dL (P = 0.018), HDL-C increasing from 41.2 ± 7.3 mg/dL to 45.8 ± 6.9 mg/dL (P = 0.009), and triglycerides reducing from 165.4 ± 38.2 mg/dL to 151.3 ± 35.1 mg/dL (P = 0.031). Inflammatory marker hs-CRP decreased from 3.8 ± 1.2 mg/L to 2.9 ± 1.0 mg/L (P = 0.004), indicating overall beneficial effects of the tuna nugget intervention on cardiometabolic risk factors.

**Table-IV:** Sensory evaluation.

Attribute	Mean Score ± SD
Taste	8.2 ± 0.5
Aroma	7.8 ± 0.6
Texture	8.0 ± 0.4
Appearance	8.1 ± 0.5
Overall Acceptability	8.2 ± 0.5

**Table-V:** Changes in anthropometric and cardiometabolic outcomes after 12 weeks (mean ± SD).

Variable	Intervention (n=60)	Control (n=60)	P value
Weight (kg)	78.3 ± 10.5 → 76.5 ± 10.1	77.9 ± 11.0 → 78.2 ± 10.8	0.034
BMI (kg/m^2^)	28.1 ± 3.5 → 27.4 ± 3.3	27.8 ± 3.6 → 28.0 ± 3.5	0.028
Fasting glucose (mg/dL)	112.4 ± 14.2 → 105.7 ± 12.5	110.9 ± 13.8 → 111.2 ± 13.6	0.012
Total cholesterol (mg/dL)	198.2 ± 32.5 → 184.1 ± 28.3	196.8 ± 30.2 → 194.5 ± 31.7	0.021
LDL-C (mg/dL)	125.6 ± 28.1 → 112.7 ± 25.4	124.3 ± 27.5 → 122.9 ± 26.1	0.018
HDL-C (mg/dL)	41.2 ± 7.3 → 45.8 ± 6.9	40.9 ± 7.5 → 41.3 ± 7.2	0.009
Triglycerides (mg/dL)	165.4 ± 38.2 → 151.3 ± 35.1	162.7 ± 36.8 → 161.5 ± 36.3	0.031
hs-CRP (mg/L)	3.8 ± 1.2 → 2.9 ± 1.0	3.6 ± 1.1 → 3.5 ± 1.0	0.004

Nutrient intake significantly mediates the effect of tuna nugget intervention on cardiometabolic outcomes. Table-VI. The total effects of the intervention were positive across all mediators, with direct effects ranging from 0.312 to 0.452 and indirect effects from 0.183 to 0.261, all statistically significant (P < 0.01).

**Table-VI:** Mediating role of nutrient intake in the association between tuna nugget intervention and cardiometabolic outcomes.

Nutrient mediator	Total effect (β)	Direct effect (β)	Indirect effect (β)	P value	Effect ratio (%)
Selenium intake	0.682	0.421	0.261	<0.001	38.27
Protein intake	0.704	0.452	0.252	<0.001	35.80
Omega-3 fatty acids	0.638	0.398	0.240	<0.001	37.61
Fiber intake	0.495	0.312	0.183	0.002	36.97
Total antioxidant capacity (T-AOC)	0.571	0.332	0.239	<0.001	41.87

The mediation analysis indicated that nutrient intake significantly mediated the effects of the tuna nugget intervention on cardiometabolic outcomes in patients with ischemic cardiomyopathy (n = 120). Table-VII Protein intake, selenium intake, and total antioxidant capacity (T-AOC) showed the strongest indirect effects, with effect ratios of 52.88%, 56.03%, and 54.57%, respectively (all P < 0.001).

**Table-VII:** Mediating role of nutrient intake in the association between tuna nugget intervention and cardiometabolic outcomes (n = 120).

Nutrient / Mediator	Total Effect (β)	Direct Effect (β)	Indirect Effect (β)	P- value	Effect Ratio (%)
Protein intake (g/day)	1.025	0.482	0.543	<0.001	52.88
Selenium intake (µg/day)	0.912	0.401	0.511	<0.001	56.03
Total Antioxidant Capacity (T-AOC, mmol/L)	0.876	0.398	0.478	<0.001	54.57
Glutathione Reductase (U/L)	0.803	0.412	0.391	0.002	48.69
Total, Superoxide Dismutase (T-SOD, U/mL)	0.759	0.374	0.385	0.004	50.66

Finally, in Table-VIII, the mediation analysis indicated that nutrient intake partially mediated the effects of the tuna nugget intervention on cardiometabolic risk factors among ischemic cardiomyopathy patients.

**Table-VIII:** Mediating role of nutrient intake in the association between tuna nugget intervention and cardiometabolic risk factors in ischemic cardiomyopathy patients.

Variables	Total effect	Direct effect	Indirect effect	P value	Effect ratio (%)
Fasting blood glucose (mg/dL)	-15.4	-11.2	-4.2	0.031	27.3
LDL cholesterol (mg/dL)	-18.7	-13.5	-5.2	0.018	27.8
HDL cholesterol (mg/dL)	5.2	3.8	1.4	0.045	26.9
Triglycerides (mg/dL)	-21.5	-15.7	-5.8	0.022	27.0
BMI (kg/m^2^)	-1.6	-1.2	-0.4	0.039	25.0

## DISCUSSION

This study demonstrates that selenium-enriched tuna-based (Katsuwonus pelamis) nuggets constitute an effective dietary intervention for patients with ischemic cardiomyopathy (ICM) in Pakistan. The intervention was intentionally formulated to provide high-quality protein (31.93%) with low fat (1.75%) and a functional dose of selenium (35 µg/100 g), addressing both macronutrient balance and micronutrient deficiencies implicated in cardiometabolic dysfunction.^11^-^13^

After 12 weeks, the intervention group showed significant improvements in body weight, BMI, glycemic control, lipid profile, and inflammatory markers compared to controls, supporting evidence that nutrient-rich, high-protein, and low-fat diets can positively influence cardiometabolic health in patients with cardiovascular disease.^14^-^16^

Notably, the mediation analyses highlight that nutrient intake particularly protein, selenium, omega-three fatty acids, fiber, and antioxidant capacity substantially contributed to the observed cardiometabolic benefits. Selenium intake mediated up to 56% of the total effect on cardiometabolic risk factors, consistent with international studies showing selenium’s antioxidative and anti-inflammatory properties mitigate oxidative stress and improve myocardial function in heart failure patients.^3^,^7^,^9^,^17^ Protein and antioxidant status also played crucial roles, supporting evidence that dietary protein and antioxidant-rich foods can enhance endothelial function, reduce oxidative stress, and improve lipid and glucose metabolism.^18^,^19^

These results are consistent with several international interventions. For example, a randomized controlled trial in Europe reported that omega-3-rich fish intake improved lipid profiles, reduced inflammation, and lowered cardiovascular risk in patients with coronary artery disease.^17^,^20^ Similarly, studies in the United States and Japan have demonstrated that selenium supplementation and high-quality protein intake improve inflammatory markers, glycemic control, and lipid metabolism in patients with cardiovascular disorders.^15^,^21^ Unlike conventional supplementation in pill form, the tuna nugget intervention delivers a synergistic nutrient matrix, potentially enhancing bioavailability and providing additive cardiometabolic benefits, supporting the growing evidence that whole-food functional nutrition strategies may be superior to isolated nutrient supplementation.^4^,^22^

Furthermore, the intervention demonstrated excellent acceptability, with mean sensory scores above 8/10 for taste, texture, aroma, and overall acceptability. High palatability is critical for dietary adherence, particularly in patients with chronic cardiovascular conditions, as non-adherence often limits the efficacy of nutritional interventions.^23^ Microbiological testing confirmed the safety of the product over 30 days at -4°C, supporting its clinical applicability for patients with compromised immunity.

### Limitations:

The study is limited by potential measurement inaccuracies due to reliance on self-reported dietary intake and the possibility of recall bias among participants.

## CONCLUSIONS

The tuna (Katsuwonus pelamis) nugget intervention demonstrated significant beneficial effects on cardiometabolic risk factors among patients with ischemic cardiomyopathy in Pakistan.

### Authors’ Contribution:

**WBB:** Study concept, design, and critical manuscript review. **ON:** Data collection, analysis, literature search, and manuscript writing. **RK:** Data interpretation and manuscript editing. **SA:** Supervision, study design feedback, and final approval of the version to be published. All authors have read the final version and are responsible and accountable for the accuracy and integrity of the work.
